# Discovery of hydroxytriazole as a potential glyoxalase-I inhibitor utilizing computer-aided drug design techniques

**DOI:** 10.1038/s41598-026-40497-4

**Published:** 2026-02-19

**Authors:** MohammedBashar Al-Qazzan, Qosay Al-Balas, Belal Alnajjar, Mohammed Al-Akeedi

**Affiliations:** 1 Department of Pharmaceutical Sciences, Faculty of Pharmacy, Al-Ahliyya Amman University, Amman, 19628 Jordan; 2 Department of Medicinal Chemistry and Pharmacognosy, Faculty of Pharmacy, Jordan University of Science & Technology, Irbid, 22110 Jordan; 3 Department of Applied Pharmaceutical Sciences and Clinical Pharmacy, Faculty of Pharmacy, Isra University, Amman, 11622 Jordan

**Keywords:** Cancer, Computer-aided drug design, Glyoxalase-I, Metalloenzyme, Molecular docking, Molecular dynamics, Virtual screening, Zinc-binding moiety, Biochemistry, Chemical biology, Chemistry, Computational biology and bioinformatics, Drug discovery

## Abstract

The glyoxalase system, particularly Glyoxalase-I (Glo-I), plays a crucial role in detoxifying aldehyde metabolites into lactic acid. Inhibiting this enzyme causes the buildup of the poisonous aldehyde, leading to programmed cell death. In this study, molecular modelling techniques were employed, including high-throughput virtual screening (HTVS), followed by filtration procedures such as Lipinski’s rule of five and Veber’s rules, and finally CDOCKER docking, to prioritize compounds from the commercial Maybridge database. Sixteen compounds were carefully chosen and purchased from the Maybridge database for further experimental evaluation. The integrated computational and experimental workflow successfully culminated in the identification of a novel, potent Glyoxalase-I (Glo-I) inhibitor. One molecule has been discovered to inhibit Glo-I with an **IC**_**50**_ **of 11.1 µM**. An analysis of the molecular dynamics of the active ligand (**SPB07393SC**) reveals stable behaviour. Crucially, this molecule incorporates a unique hydroxy triazole moiety, representing the first reported instance of this zinc-coordinating group in a Glo-I inhibitor. This will be utilized for the purpose of developing novel compounds with enhanced activity following appropriate modifications.

## Introduction

Cancer is the unregulated growth of cells that spreads through the entire body^1^. Despite continued attempts to combat it, cancer remains a major health issue worldwide^2^. It is therefore critical to identify novel medications and to study cancer behaviour to identify valid targets. Glo-I is a promising target that can be selectively blocked with a high likelihood of success^3–5^.

**Fig. 1:**
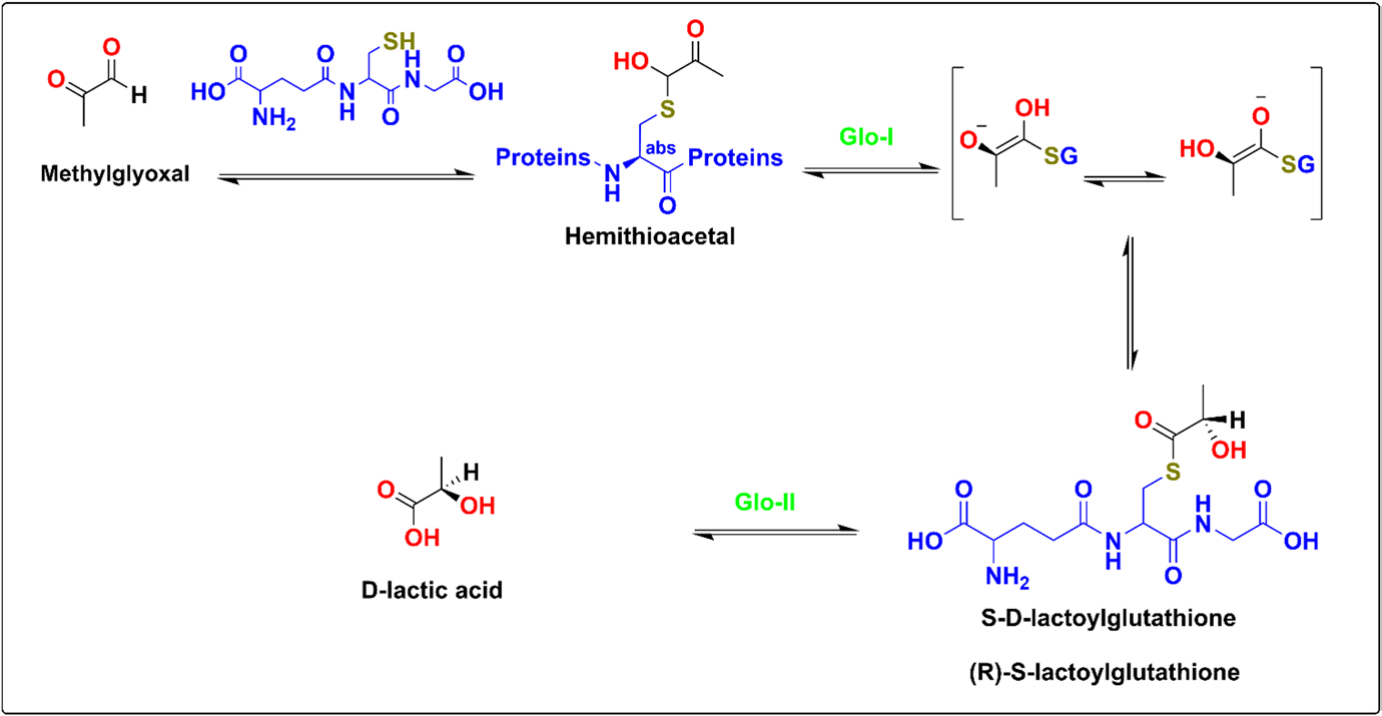
Detoxification process of methylglyoxal via the glyoxalase system.

The glyoxalase system is an essential cellular mechanism that detoxifies metabolic byproducts, such as methylglyoxal, formed during glycolysis, into the non-toxic D-lactic acid, as shown in Fig. 1^6–9^. The system consists of two enzymes, glyoxalase I (Glo-I) and glyoxalase II (Glo-II), and a catalytic quantity of glutathione (GSH). Notably, blocking the glyoxalase pathway, specifically Glo-I, in cancer cells has been proposed to lead to the accumulation of methylglyoxal, ultimately causing cancer cells to self-destruct^10^.

**Fig. 2:**
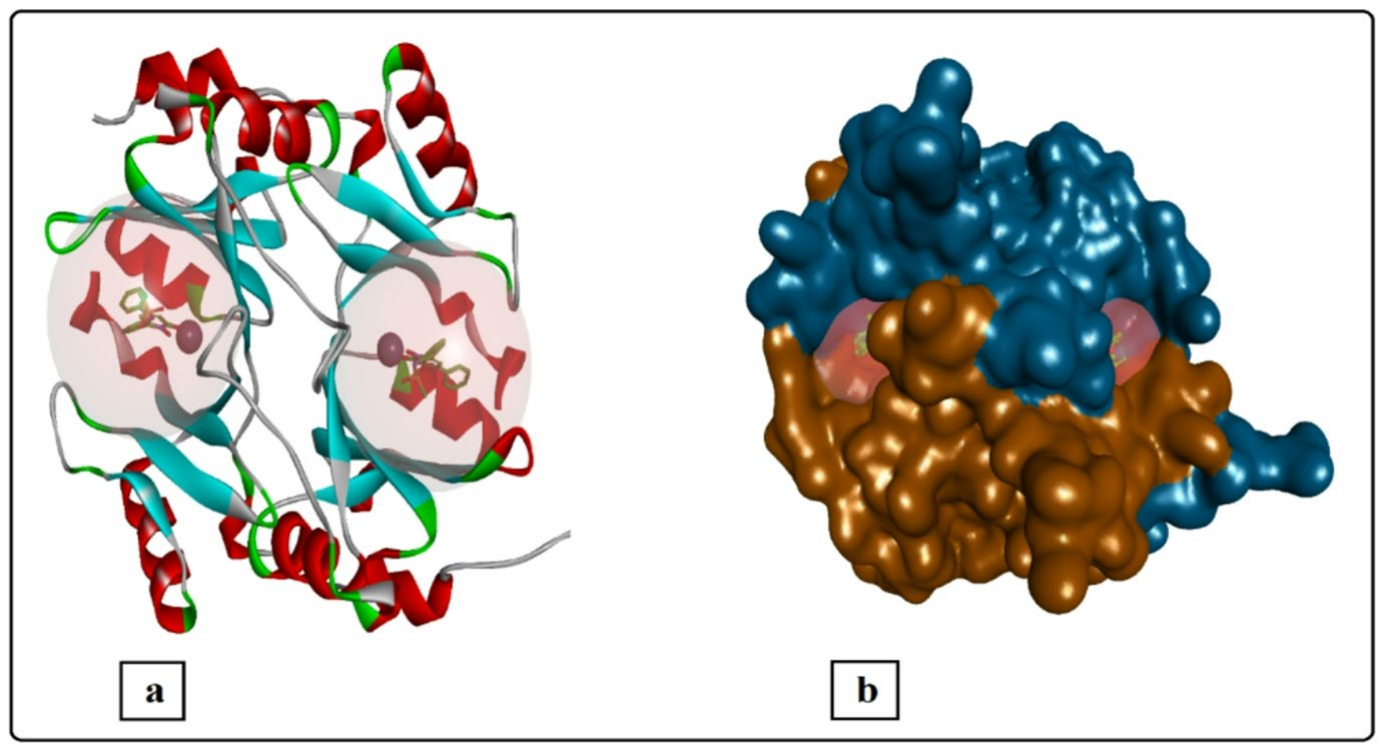
The crystal structure of human Glo-I (PDB code 3W0T). (**a**) Schematic representation of the human Glo-I structures (PDB code 3W0T) in cartoon representation, the ligand is shown in balls and sticks and Zn + 2 ions as CPK style gray spheres. (**b**) Surface representation of the human Glo-I structure showing the location of the binding sites at the interface of the two homodimers.

The human Glo-I is a homodimeric, mononuclear zinc metalloenzyme with a mass of 42 kDa and 184 amino acids in each monomer^11^. It has been established that Glo-I contains two identical active sites located at the dimer interface on opposite sides (Fig. 2). According to the crystal structure, the active site can be divided into three sections: a positively charged entrance, a central zinc atom, and a deep lipophilic pocket^12^.

The most crucial region is the zinc-binding site at the base of the active site. Three amino acids-Glu99, Gln33, and His126-are coordinated with the zinc atom in these molecules. It is significant to note that zinc ions frequently exhibit a coordination number of four and a tetrahedral arrangement of ligands in protein structures. This allows the zinc atom to form an additional coordination bond with the zinc-binding group of the inhibitor^13,14^. When developing Glo-I inhibitors, this feature must be considered. In the Glo-I enzyme, a tiny hydrophobic pocket that can contain up to two aromatic rings serves as the second binding site. The amino acids Leu92, Phe71, Met179, Leu160, Leu69, and Phe62, which are all hydrophobic, make up this hydrophobic region. A highly polar binding region is also present in the enzyme, located near the entrance to the active site. It is made up of the positively charged side-chained polar amino acids Arg122, Arg37, Lys150, and Lys156^11,15,16^.

Although several computational and experimental studies have targeted Glo-I, most reported approaches have so far relied on small-scale virtual screening or limited chemical diversity. Others have focused mainly on classical zinc-binding groups such as hydroxamates, carboxylates, or catechols. However, these scaffolds often show metabolic instability or poor selectivity. This underscores the need to explore alternative chemotypes with improved zinc-coordination behavior.

In the present study, we applied an integrated computer-aided drug design workflow. This workflow combines HTVS, physicochemical filtration, molecular docking, and molecular dynamics simulations. We systematically explored the broad chemical space of the Maybridge Screening Collection. Our approach was instrumental in identifying nonclassical zinc-binding moieties and novel ligand-protein interaction profiles, which previous Glo-I optimization efforts did not thoroughly address.

Importantly, the molecular modelling techniques used here provide significant predictions of the key structural features responsible for zinc coordination. They also estimate the stability of binding within the dynamic enzyme pocket and allow ranking of compounds with favorable pharmacokinetic behavior before experimental assessment. This in silico study is expected to yield structurally distinct new Glo-I inhibitors. These may serve as promising starting points for further lead optimization and the development of anticancer agents.

## Materials and methods

### Experimental materials and computational software tools

Ligand and protein preparation and molecular docking (LibDock, CDOCKER) were done using *BIOVIA Discovery Studio*. IC50 values were determined by nonlinear regression in *GraphPad Prism*. *GROMACS* was used for molecular dynamics simulations. MD trajectory plots for root-mean-square deviation (RMSD), Root Mean Square fluctuation (RMSF), hydrogen bond count(H-bonds), radius of gyration (Rg), and solvent-accessible surface area (SASA) were generated with XMGrace.

Experimental materials included human recombinant GLO-I (rhGLO-I) and coli-derived Ala2-Met184 with an N-terminal Met and 6-His tag (R&D Systems Corporation, USA) to test the inhibitory activity of the chosen compounds against GLO-I in vitro. Final compounds selected via virtual screening were purchased from the Maybridge and Aldrich databases through local suppliers (*Maybridge Chemical Holdings Ltd.*,* a UK brand of Thermo Fisher Scientific*). A Plate Reader (SpectraMax Plus, Molecular Devices) assessed the biological activity of selected compounds in vitro. Sigma-Aldrich reagents included deionized water, sodium phosphate (monobasic and dibasic), DMSO, reduced GSH, and methylglyoxal solution.

### Computational approaches

#### GLO-I crystal structure preparation

Stability and flexibility properties of the protein-ligand complex were obtained from MD simulations. Specifically, **SPB07393SC** exDS was utilized to prepare structural models of the human GLO-I enzyme. To acquire the 3D coordinates for the GLO-I enzyme^17^, direct access to the RCSB Protein Data Bank was used. There are six Glo-I protein crystal structures in humans under the following entry codes: 1QIN^18^, 1QIP^18^, 3W0T^19^, 3W0U^19^, 1FRO^20^, and 3VW9^19^. For this project, one crystal structure with the PDB code **3W0T (resolution of 1.35Å)** complexed with N-hydroxypyridone derivative inhibitor was selected as the working model^19^. The Protein Report program was then used to examine the crystal structure for missing loops, alternative conformations, and incomplete residues. Although the simulation initially showed a high degree of fluctuation, it eventually stabilized, indicating that the ligand indeed binds well to Glo-I. Furthermore, the simulation data confirm that **SPB07393SC** maintains efficient interactions with the enzyme’s active site. This is supported by the ligand’s unique structural features, including the triazole and coumarin rings, which could stabilize the enzyme-ligand complex through coordination to the zinc atom.

The “Prepare-Protein” protocol was subsequently employed to clean each structure, removing any water molecules or other non-protein atoms, optimizing the hydrogen bonding network, standardizing atom names, adjusting connectivity and bond orders, and simulating proteins at pH 7.4 (± 0.2), after which the co-crystallized ligands were removed. This can be accomplished by using Discovery Studio’s “Protein Preparation” tools^21,22^. To prepare all structures for subsequent handling by other protocols, the Simulation Tools were used to type each structure using the CHARMm force field. The complex was initially solvated using the Solvate procedure, with a minimum distance from the cell boundary of 5.0 Å, by submerging it in a pre-equilibrated truncated octahedral box of explicit water. The solvated system was then minimized using the smart minimizer within the Minimization protocol over three successive phases.

During the first minimization step, the heavy atoms of the protein and complexed ligand were subjected to a harmonic restraint. In the following step, only the backbone atoms were restrained. At the final stage, all restraints were removed, and the system was allowed to move. After minimization, water molecules were removed using the “Define and Edit Binding Site” tool. The co-crystallized ligand (HPU) was used to define the binding site and was then removed. The generated sphere’s radius was increased to 12.5 Å to include all binding site areas.

#### Preparation of ligands

The Maybridge Screening Collection (2021) was downloaded in SDF format, containing 53,352 compounds to ensure chemical diversity within the constraints of available computational and experimental resources^23^. Larger libraries would exceed the available resources and project timelines. Once downloaded, the compound library was prepared for virtual screening using the “Prepare Ligand” tool. This protocol performs tasks such as optimizing ligand conformations, cleaning up the structures, removing duplicates or problematic compounds or salts, generating possible states at the target pH 7.4 (± 0.2), correcting protonation states, assigning 3D coordinates, and generating tautomers. The default values for all parameters were used, except for the ionization technique, which was set to rule-based. The tautomerization method was set to the canonical tautomer, and the isomerization method’s value was set to false.

The Maybridge Screening Collection contains 53,352 compounds, split using the “Split Ligands” protocol into 10 sections. The preparation of ligands for each split has doubled the total number to approximately 10,862 ligands. The energy of ligands was minimized using the CHARMM force field and the smart minimizer algorithm with default values, followed by the steepest descent and conjugate gradient algorithms until the compounds reached a convergence gradient of 0.001 kcal/mol. After generating 3D structures and performing minimization, the compounds are ready for virtual screening and ligand docking.

### Docking validation

**Fig. 3:**
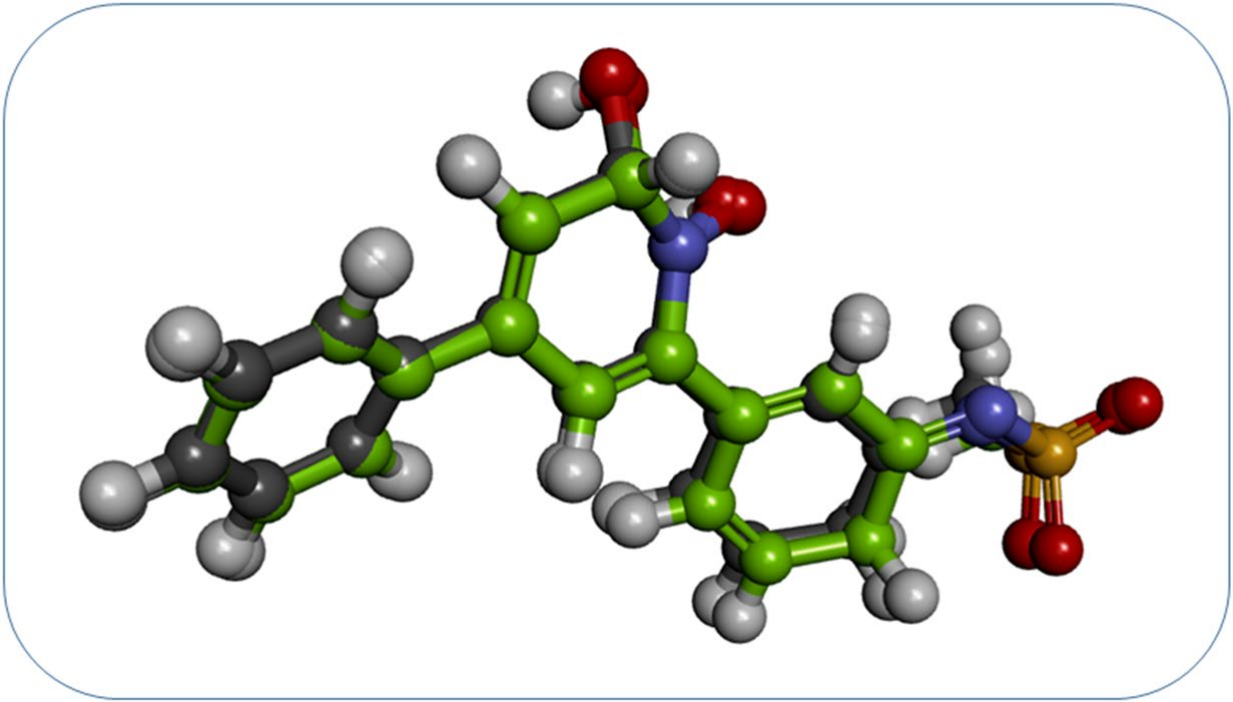
The best orientation of overlap between the docking pose (black colour) from the PDB Code:3W0T and the ligand pose from the crystal form (green colour).

The enzyme is now ready for docking. It is important to verify the validity of the intended docking parameters by examining the orientation of the co-crystallized ligand after docking. To do this, copy the co-crystallized ligand, prepare it, and re-dock it to observe its binding pattern and docked pose^24^. The resulting pose has an RMSD of 0.2558Å, as shown in Fig. 3. An RMSD value of less than 2Å indicates an acceptable overlap; lower values indicate more similarity and a better fit of the ligand in the active site.

### Virtual screening

**Fig. 4:**
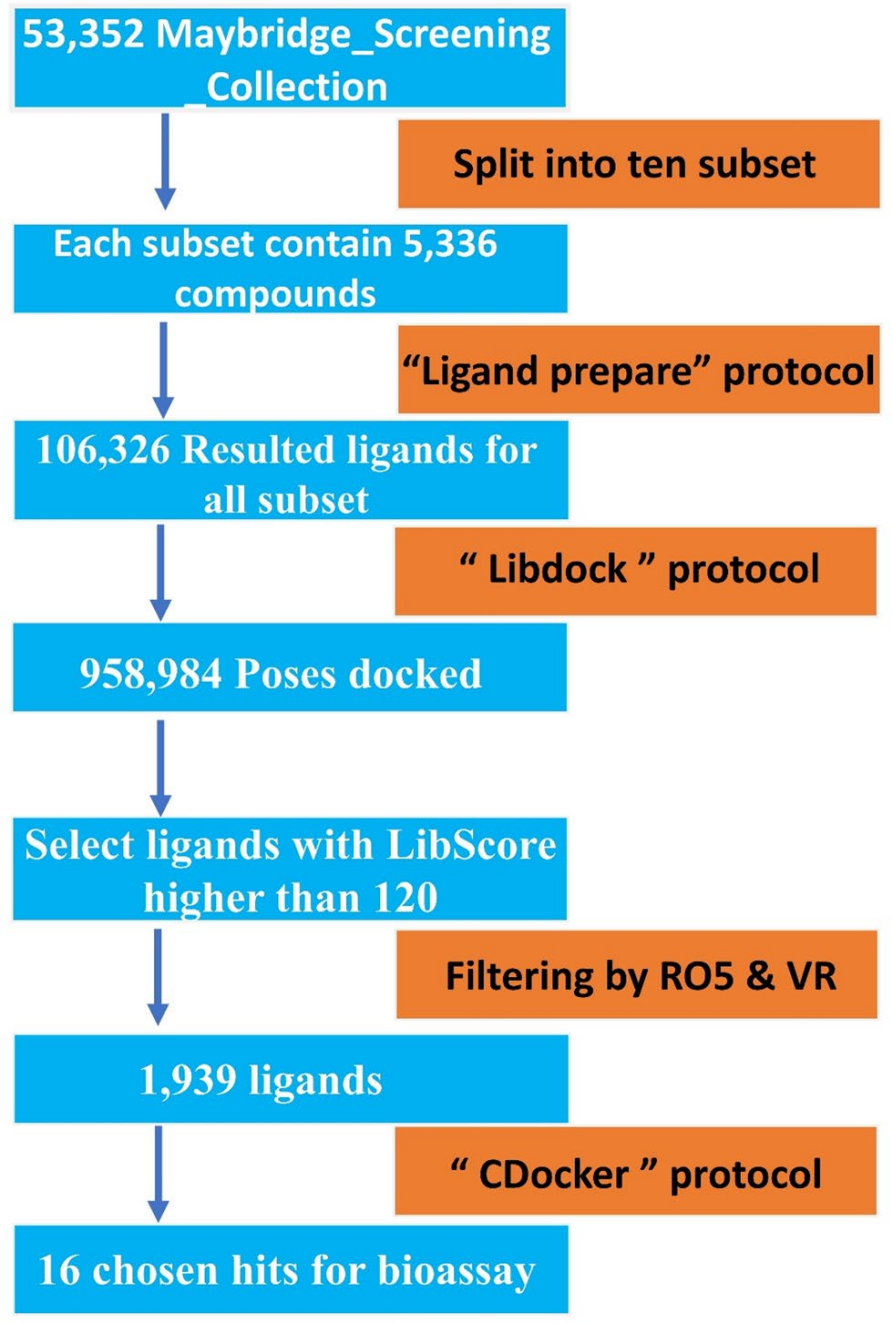
Hit identification process from HTVS to molecular docking, followed by final hit selection based on docking scores and molecular properties.

The virtual screening process began by using the LibDock protoco^25,26^. This is a fast search method that enables us to prioritize ligands with high scores for later use with CDocker. The cut point used to select compounds from LibDock to CDocker was set to 120, as specified in the LibDock protocol. Compounds were then subjected to a more robust, extensive search using CDocker^27,28^. The whole computational procedure for hit identification is shown in Fig. 4.

### Molecular docking

To determine the interaction energy and the ideal orientation between the target protein and ligand molecules, molecular docking was performed, using the prepared Glo-I crystal structure with the best resolution (PDB code: 3W0T, resolution of 1.35Å). To dock ligands into a receptor binding site, a high-throughput site-featured algorithm was used with the docking software LibDock(hotspot-directed- high throughput docking), which is provided by DS with default parameters except for docking performance (Fast search), conformation method (Fast), and minimization algorithm (do not minimize). When the ligand aligned to create a favourable interaction, LibDock used features of protein polar and apolar interaction sites, known as hot spots. Default values for all additional docking and scoring parameters were selected^29,30^.

The best pose has been selected based on interactions with the protein’s significant residues and the interaction energy between the ligand and the protein. Each pose was assessed using the LibDock score, calculated using a simple pairwise technique, and ligands with LibDock scores of 120 or higher were selected. To improve the drug-likeness of our screening results, the Maybridge Screening Collection was filtered using the Lipinski rule of five and the Veber rule in DS 2022. Next, the filtered Maybridge Screening Collection was further submitted to molecular docking using the CDocker tool (CHARMm-based docking). The protocol had been implemented with the default parameters except for Force field (CHARMm) and Ligand partial charge Method (Momey-Rone)^31,32^. The top 50 compounds of each split were investigated visually.

### Final hit selection

Following completion of all filtering steps, compounds were selected based on their CDocker scores, the chemical structures of the hits, and visual analysis of docked pose alignments.

### Molecular dynamics simulation

A 100 ns Molecular Dynamics (MD) simulation was performed using *GROMACS*-2023.1 for both our selected hit compound and the native ligand targeting human Glo-I (**PDB ID: 3W0T**). The protein topology was generated with the CHARMM36 force field. Ligand topology was prepared using the General force field (CGenFF) server. Solvation employed a dodecahedral unit cell. Periodic boundary conditions were used, with a 10 Å cutoff to prevent interactions beyond the box edges^33^.

Ions were introduced using the steepest descent minimization algorithm, with sodium and chloride ions used to neutralize the protein. Next, the complex underwent energy minimization to alleviate steric clashes, employing the steepest descent minimization algorithm with a force cutoff of 10.0 kJ/mol and a maximum of 50,000 steps. Following minimization, two equilibration processes, NVT and NPT, were performed using a modified Berendsen thermostat and leap-frog integrator for 50,000 steps each, equivalent to 10 ps. Finally, the MD simulation was conducted for 50 ns, with a 2-fs time step.

## Experimental approach (biological evaluation)

### In vitro enzyme bioassay

The inhibitory activity of selected hits against GLO-I was assessed in vitro using recombinant human GLO-I (rhGLO-I)^34,35^. Each selected hit was tested in three separate experiments, each in triplicate using a plate reader, and the averages were calculated. IC50 values were determined using *GraphPad Prism* 8 (2019). The enzyme was reconstituted in our facility by dissolving 0.5 mg/mL in sterile deionized water, then stored at -70 °C and thawed at 7 °C in the refrigerator on the test day. Subsequently, selected hits were dissolved in DMSO to make a 10 mM standard solution. For assay buffer preparation, a 0.5 M stock solution of sodium phosphate dibasic (solution 1) was made by dissolving 35.5 g in distilled water to a final volume of 500 mL.

Furthermore, 0.5 M sodium phosphate monobasic stock solution (solution 2) was created by dissolving 30 g of anhydrous sodium phosphate monobasic in 500 mL of distilled water. The next step was to dilute 80 mL of stock solution 1 in 400 mL of distilled water to create a 0.1 M sodium phosphate dibasic solution (solution 3). Additionally, a 0.1 M sodium phosphate monobasic solution (solution 4) was prepared by diluting 30 mL of stock solution 2 with water to a final volume of 150 mL. Finally, solution 4 was added to solution 3 until the pH reached 7.4 (± 0.2), ensuring optimal conditions for enzyme activity.

### Glo-I inhibition procedure (plate reader)

Heat the water bath to 37 °C. To prepare the glutathione stock solution (100 mM), dissolve 37.16 mg of glutathione in 1200 µL of deionized water. Mix until fully dissolved. Next, prepare a methylglyoxal solution (100 mM); once prepared, pipette 18.48 µL of this solution and add it to 1200 µL of buffer. Mix for 15 s, then vortex. Prepare a stock solution of each test compound in DMSO, then dilute each to the necessary concentration using buffer. To prepare the substrate solution, mix 25 mL of buffer with 706 µL of methylglyoxal solution and 706 µL of glutathione stock solution. Vortex this mixture for 15 s. Finally, incubate the substrate solution in the 37 °C water bath for 15 min.

**Fig. 5:**
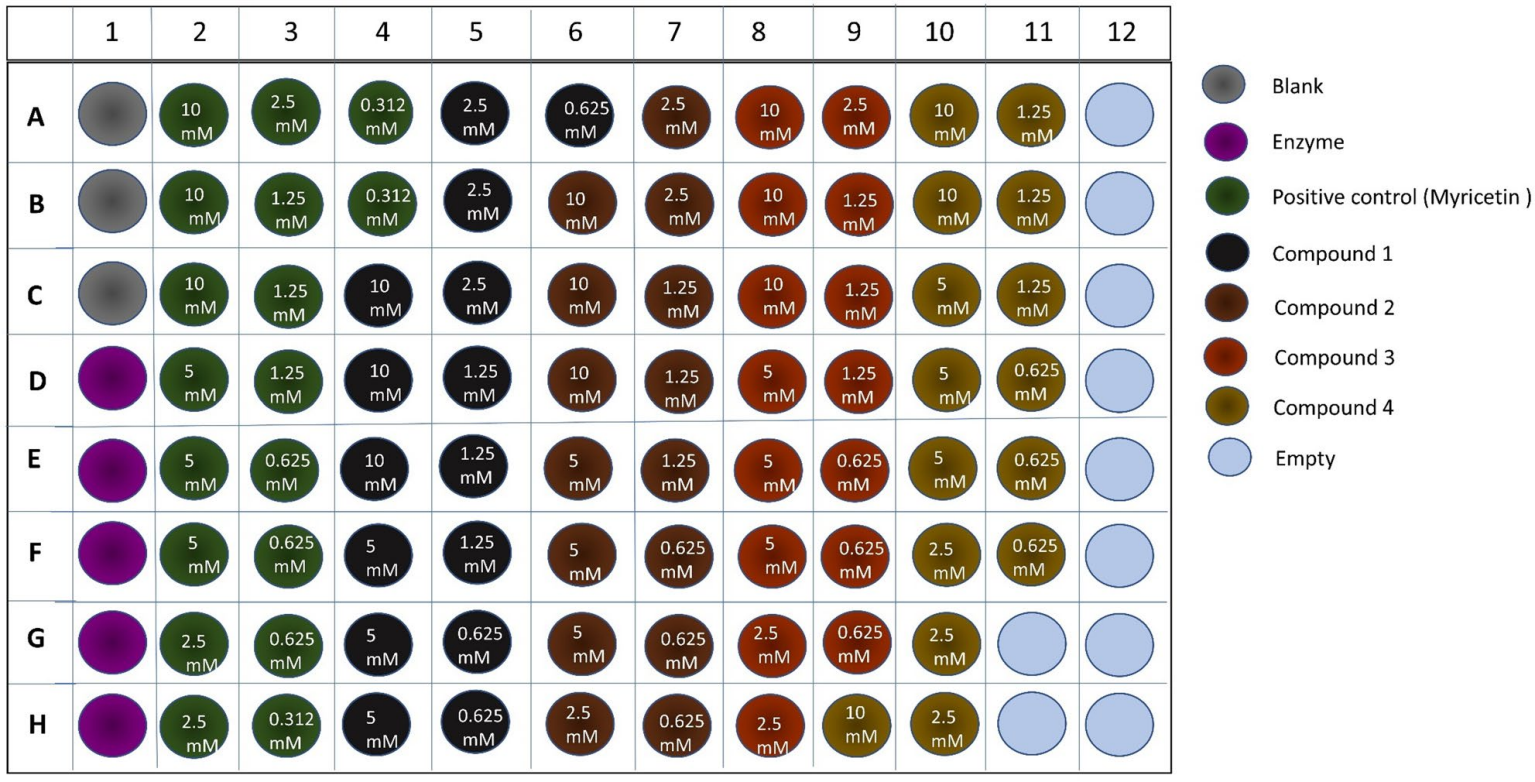
Schematic representation of the 96-well plate setup for the Glo-I bioassay, used to assess enzyme activity across various test samples.

Incubate two tubes of buffer (10 mL) in the same water bath. Add 1 µL of DMSO to both the blank wells and the enzyme activity wells (column 1). For all other wells, add 1 µL of the specific compound assigned to each well, as shown in Fig. 5^35,36^. Prepare the enzyme as follows: combine 17.75 µL of enzyme with 10 mL of incubated buffer (step 8). Add 49 µL of buffer to the blank wells and 49 µL of enzyme to all wells except the blank wells. Incubate the plate at 37 °C for the desired amount of time. Using a Synergi 2 microplate reader, read the absorbance in kinetics mode.

### Result interpretation

After performing the assay and obtaining the absorbance values, percentage inhibition and IC50 can be calculated as follows:

At the beginning, for each well, determine the absorbance value at 0 and 200 s from the plate reader. By subtracting the average absorbance value of the blank wells from the absorbance value of the corresponding enzyme at 0 and 200 s. And then subtract the average absorbance of blank wells at 0 and 200 s from the absorbance of the compound well at 0 and 200 s. Then calculate the percentage inhibition of the enzyme by the compound in each well using the following formula:

$$\% inhibition = \left( {1 - ~\frac{{absorbance~of~the~compound~at~200\sec ~ - ~absorbance~of~the~compound~at~0\sec )}}{{absorbance~of~the~enzyme~at~200\sec ) - absorbance~of~the~enzyme~activity~at~0\sec )~}}~~} \right)*100$$

Finally, Plot the log concentration of the compound against the average % inhibition of the enzyme from all wells using *GraphPad Prism* to obtain the IC_50_.

## Results and discussion

### Molecular docking

The docking process started with a two-stage strategy. First, there was a quick LibDock prioritization step. This was followed by an extensive final compound selection in CDocker. The LibDock protocol parameters were left at their defaults, except that “Docking performance” was set to fast. No minimization steps were allowed. The resulting docked ligands totaled 106,326, with 57 failed ligands. The reason for this fast search protocol was to quickly prioritize ligands with high binding capacity. This approach saves time and reduces power consumption at workstations.

The ligands with scores over 120 were selected to proceed to the next stage after docking with CDocker. The reason behind choosing “fast search” is that the observation of most ligands identified by the “fast search” protocol are also identified by the “high quality” protocol, but the only difference is the arrangement of the ligands in the output, which is not matter now, because the ligands then will be assigned to more robust software, which is CDocker. The total ligands passed this stage were 958,984, which were then sent for further drug likeness filtration by employing Lipinski’s rule of five and Veber’s rules. By filtering the top compounds with a LibDock score of more than 120 using Lipinski’s rule of five (RO5) and Veber’s rules (VR), it’s likely narrowed the list to compounds with a higher likelihood of being orally bioavailable and a better pharmacokinetic profile. These compounds were further evaluated using CDocker software.

After applying the Lipinski Rule of Five to the ligand library, the qualifying ligands were evaluated using the CDocker docking algorithm, with various parameters set to optimize the process. After CDocker simulations, ligands were assessed based on binding scores. Ligands with high scores, indicating good binding potential, underwent further analysis of chemical properties, stability, and solubility. Ligands with low scores, considered to have low binding potential, were excluded from further evaluation.

### Selection criteria

Once docking was completed by CDocker, it is important to note that ligand selection was not based solely on CDocker’s binding top scores, but also on the binding pattern within the active site, which was investigated by manual inspection^37,38^. After studying the Glo-I enzyme, three key factors that influence the binding of possible inhibitors were identified.

First, the zinc atom located at the centre of the active area, which forms coordination bonds with the prospective inhibitor, is considered the most important binding^27,39^. The second important factor to consider when choosing Glo-I inhibitors is the hydrophobic pocket next to the zinc atom, and finally, the positively ionized entrance. The final compounds refined and selected are shown in Table 1, along with their structures and the expected zinc-binding groups^40^.

**Table 1:** Final selected compounds along with their in vitro enzyme bioassay score.

Code	Structure	CDocker interaction energy(kcal/mol)	Percent of inhibition % (50µM)	IC_50_
#1 HTS06312SC		77.8374	13.13	*ND
#2 BTB15187SC		69.5134	1.22	ND
#3 RJC03488SC		64.1583	40.53	ND
#4 SPB08317SC		64.0228	22.06	ND
#5 SCR00479SC		61.7253	-10.44	ND
#6 RH011833SC		60.7722	8.95	ND
#7 BTB13329SC		60.5258	13.58	ND
#8 HTS11145SC		58.5385	9.33	ND
#9 LT00645585		57.8721	18.75	ND
#10 HTS07045SC		56.7722	5.09	ND
#11 SEW02685SC		55.1326	-16.49	ND
#12 LT00724248		49.918	-8.39	ND
#13 SPB07393SC		48.2166	78.66	11.1µM
#14 JFD02279SC		47.4819	6.43	ND
#15 BTB02024SC		46.8452	8.34	ND
#16 BTB13866SC		44.9216	-8.33	ND
Positive control (Myricetin)		39.7299	106.92	3.6 µM

^*ND= Not Determine^.

**Fig. 6:**
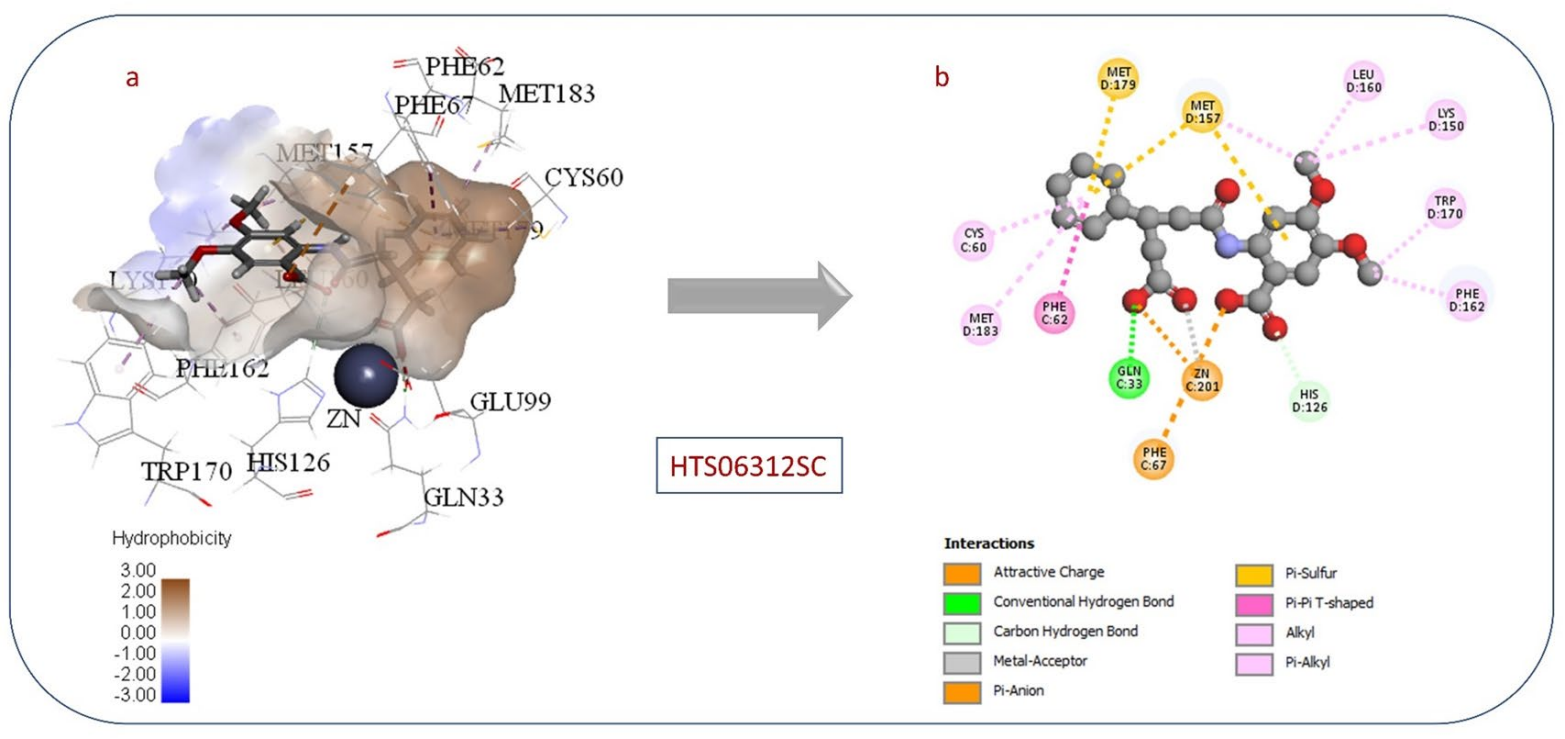
(**a**) 3D view of (**HTS06312SC**) in Glo-I active site. (**b**) 2D interactions between (**HTS06312SC**) and Glo-I active site.

Compound HTS06312SC had the highest CDocker interaction energy, 77.8374, and its interaction pattern with the active site is shown in Fig. 6. Interestingly, both carboxylates participate in coordination to the zinc atom. The phenyl ring occupies the hydrophobic pocket next to the zinc atom by forming interactions with nonpolar amino acids, such as Met 183 and Cyc 60. The polar active site has also contributed to ligand binding by forming ion-dipole interactions with Gln 33. The methoxy groups both expressed hydrophobic interaction with the hydrophobic side chains of Phe162 and Trp170.

**Fig. 7:**
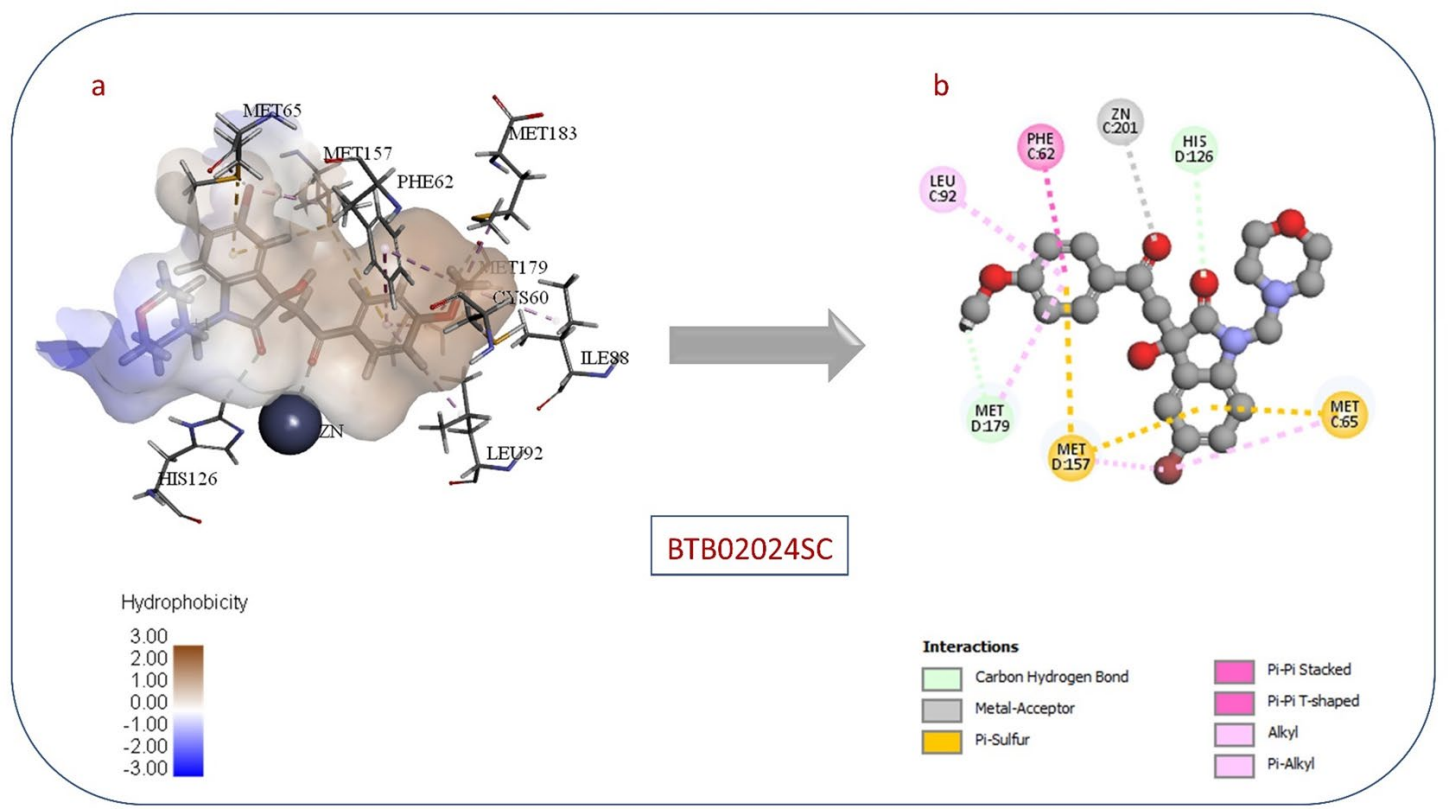
(**a**) 3D view of (**BTB02024SC**) in Glo-I active site. (**b**) 2D interactions between (**BTB02024SC**) and Glo-I.

Compound BTB02024SC had a CDocker interaction energy of 46.8452, and its interactions with the active area are shown in Fig. 7. Notably, the ketone group participated in coordination with the zinc atom, highlighting a key binding interaction. In addition, the phenoxy ring occupies the hydrophobic pocket near the zinc atom by interacting with nonpolar amino acids, including Leu92, Met157, and Phe62.

**Fig. 8:**
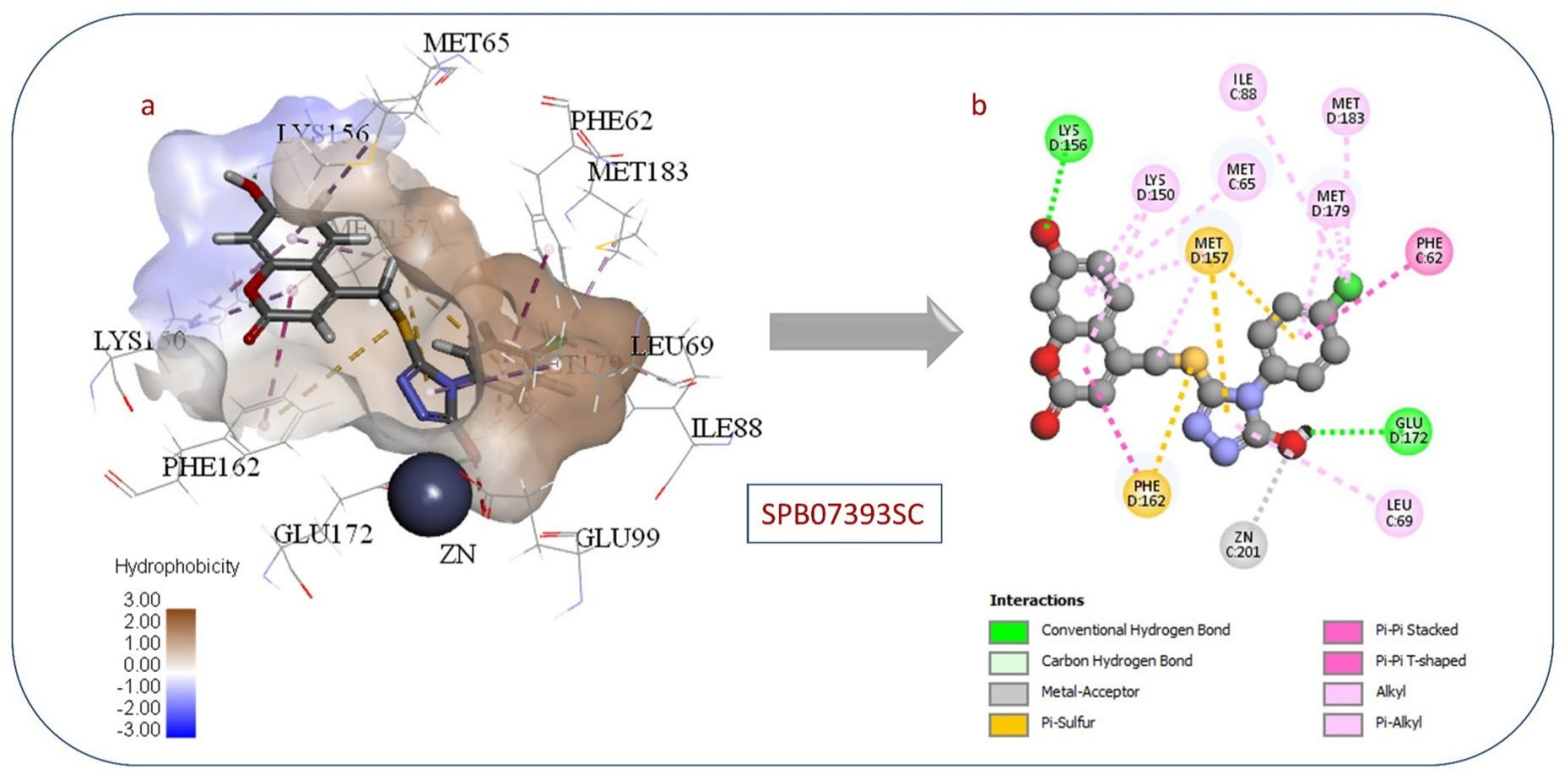
(**a**) 3D view of (**SPB07393SC**) in Glo-I active site. (**b**) 2D interactions between (**SPB07393SC**) and Glo-I.

Compound **SPB07393SC** has a CDocker interaction energy of 48.2166. Its interaction with the active area is shown in Fig. 8. Interestingly, the hydroxyl group on the triazole ring coordinates to the zinc atom. The benzene ring fills the hydrophobic pocket next to the zinc atom. This occurs through interactions with non-polar amino acids, including Pi-Pi stacking with Phe 62. Chlorine exhibits hydrophobic interactions with Met 183, Ile 88, and Met 179. The polar active site Lys 156 forms a H-bonds with the hydroxyl group on the coumarin ring. Pi-pi stacking interactions also occur between the hydrophobic side chains of Phe162 and the coumarin ring.

**SPB07393SC**’s binding affinity stands out due to its hydroxytriazole moiety, a group not previously implicated in Glo-I inhibition. This moiety coordinates effectively with the enzyme’s active-site zinc atom, enabling inhibition. Importantly, the zinc-coordinating group offers potential for greater selectivity and stability in Glo-I inhibitor development. It may also address the poor metabolic stability observed with common scaffolds such as hydroxamates and carboxylates.

### In silico pharmacokinetics prediction for the chosen active compound

The ADMET properties of the active compound **SPB07393SC** were evaluated, including Lipophilicity (clogP), polar surface area, molecular weight (MW), and primarily aqueous solubility (logS). In addition to evaluating its metabolism via CYP2D6, the prediction of absorption level, plasma protein binding (PPB), the ability of the compound to cross the blood-brain-barrier (BBB), and hepatotoxicity.

(Table 2) shows the PK profile of **SPB07393SC**. It has a good level of solubility (level 2) and good penetration across the blood-brain barrier (BBB) (score = 4), indicating good penetration. Its AlogP value (Lipophilicity) is 4.395, which applies Lipinski’s rule of five for oral bioavailability. The predicted absorption level is zero, indicating good absorption ability. The CYP2D6 probability score is -0.910579, indicating that it cannot be an inhibitor of the CYP2D6 enzyme.

**Fig. 9:**
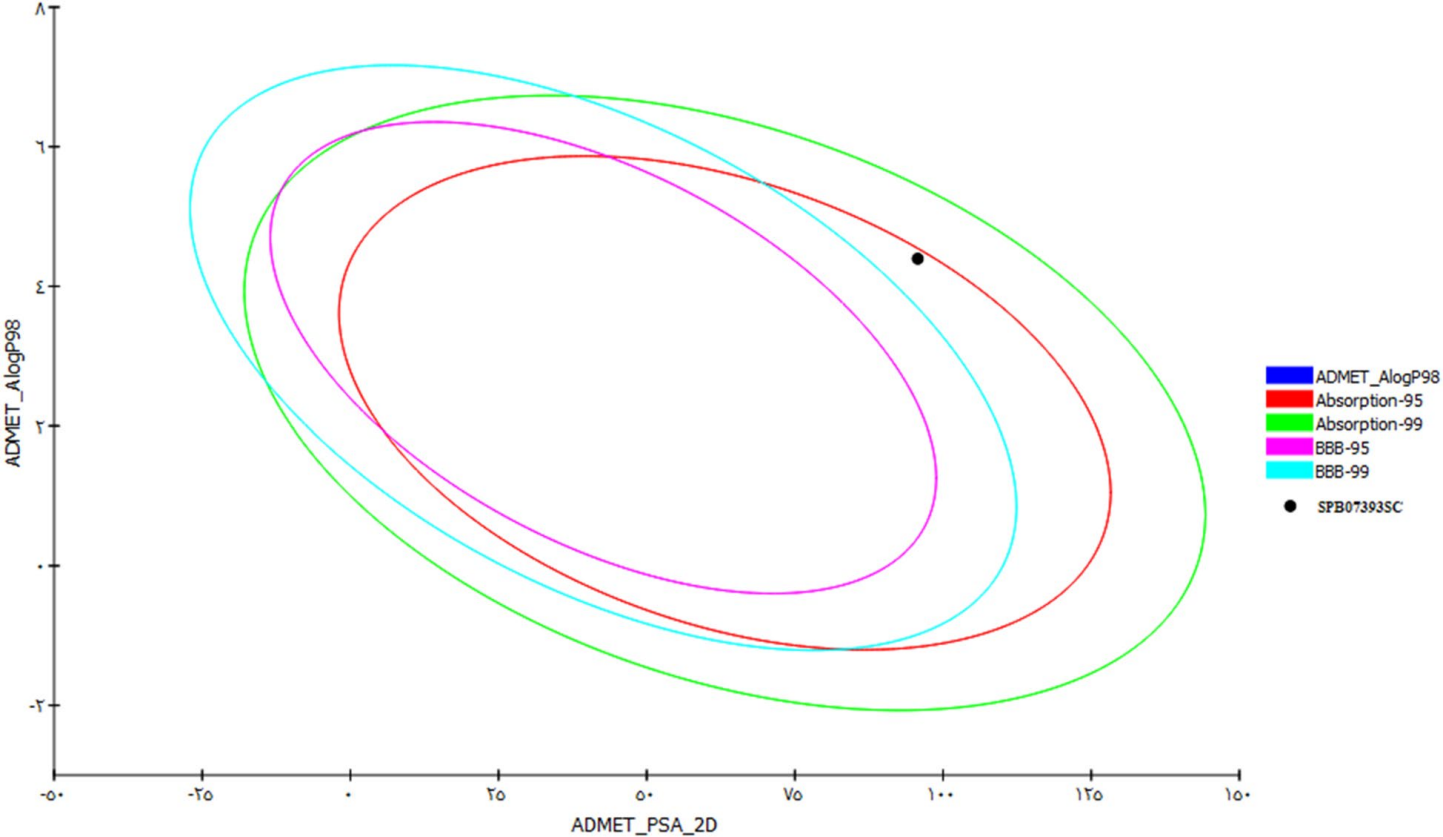
ADMET profile of the active compound (**SPB07393SC**), illustrating key pharmacokinetic properties.

The toxicity of **SPB07393SC** was assessed using the TOPKAT tool in Biovia DS. **SPB07393SC** shows hepatotoxicity and an Ames test result of less than 7, indicating it is non-mutagenic and has a safe profile. All chosen compounds are illustrated in the ADMET plot in Fig. 9.

**Table 2:** Pharmacokinetics profile of the active choseen compounds.

Code	ADMET BBB level	AlogP	Solubility level	Absorption level	PPB	CYP2D6	Hepatotoxicity	Ames-prediction
**SPB07393SC**	4	4.395	2	zero	2.132	-0.910579	3.01181	**NM

**Non- Mutagen.

ADMET prediction provided the pharmacokinetic profile of **SPB07393SC**. The compound showed favorable properties. It passed Lipinski’s rule of five (RO5), had an AlogP value of 4.395, indicating moderate lipophilicity, and was predicted to have good solubility and absorption. **SPB07393SC** also penetrated the BBB well, making it a useful compound for systemic therapies. Additionally, minimal interaction with CYP2D6 may reduce the risk of adverse drug-drug interactions.

These pharmacokinetic data are important for developing **SPB07393SC** as a lead compound. Favorable BBB penetration enables testing in CNS-related cancers or diseases where Glo-I plays a role. The safe toxicity profile—non-mutagenic and non-hepatotoxic—suggests that **SPB07393SC** is not only a good drug candidate but also likely to cause fewer side effects, a key advantage in therapy.

### Molecular dynamics

The conformational alterations within the protein-ligand complex were investigated using two methodologies: RMSD and analysis of SASA over a 100 ns MD simulation, aiming to evaluate the stability of the simulated system. These metrics were computed after re-centering and re-wrapping the complex within the unit cells using *GROMACS’s* trjconv function.

**Fig. 10:**
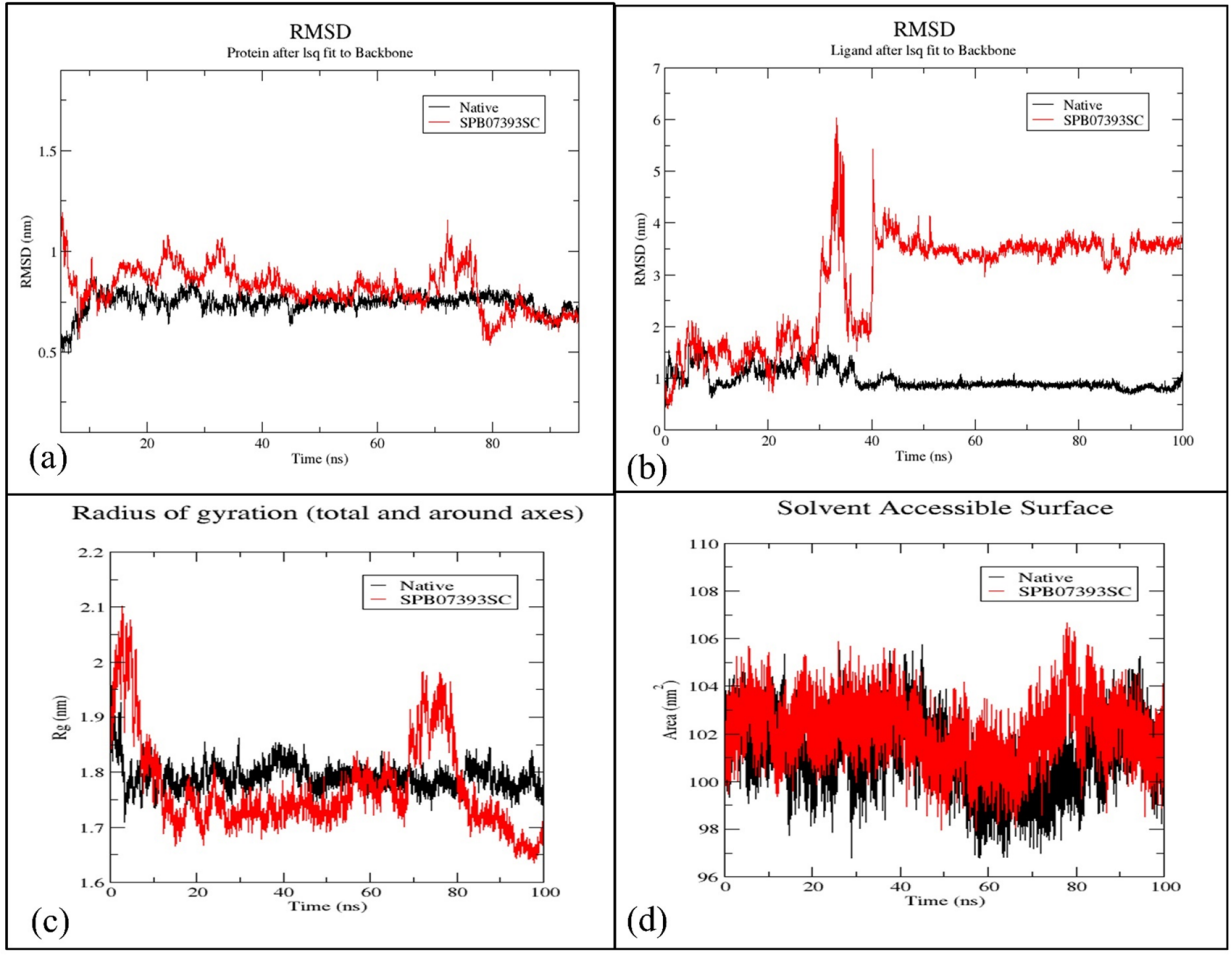
Structural dynamics of Glo-I RMSD (**a**), ligands RMSD (**b**), Glo-I radius of gyration (**c**), and SASA values (**d**) calculated during the 100 ns of MD trajectories.

The RMSD graph (Fig. 10a) provides insights into the stability of Glo-I and its native ligand binding. It depicts conformational variations observed throughout the 100 ns MD simulation. Notably, the backbone’s RMSD exhibits consistent fluctuations, indicating a stable trajectory for the co-crystallized ligand with minimal oscillation. The ligand **SPB07393SC** demonstrates relatively stable behavior with modest oscillation. This ligand shows a slight increase in fluctuation between 70 and 80 ns, but stabilizes during the final 20 ns. Comparable RMSD patterns are observed for the ligands upon their interaction with the target protein (Fig. 10b). While the native ligand exhibits minimal fluctuation, **SPB07393SC** gradually stabilizes after 40 ns from the simulation’s onset. It then displays a stable fluctuation of approximately 0.1 nm, well within an acceptable range of stability^41,42^.

The compactness of the protein backbone in both complexes was evaluated using the Rg over the simulation (Fig. 10c). When bound to the native ligand, the protein backbone was more compact, indicating higher stability. With **SPB07393SC**, compactness improved overall, with a spike between 70 and 80 ns, which aligns with RMSD findings. SASA analysis further supported stability, showing low, consistent fluctuation (98–105 nm²) across all three complexes throughout the simulation (Fig. 10d).

**Fig. 11:**
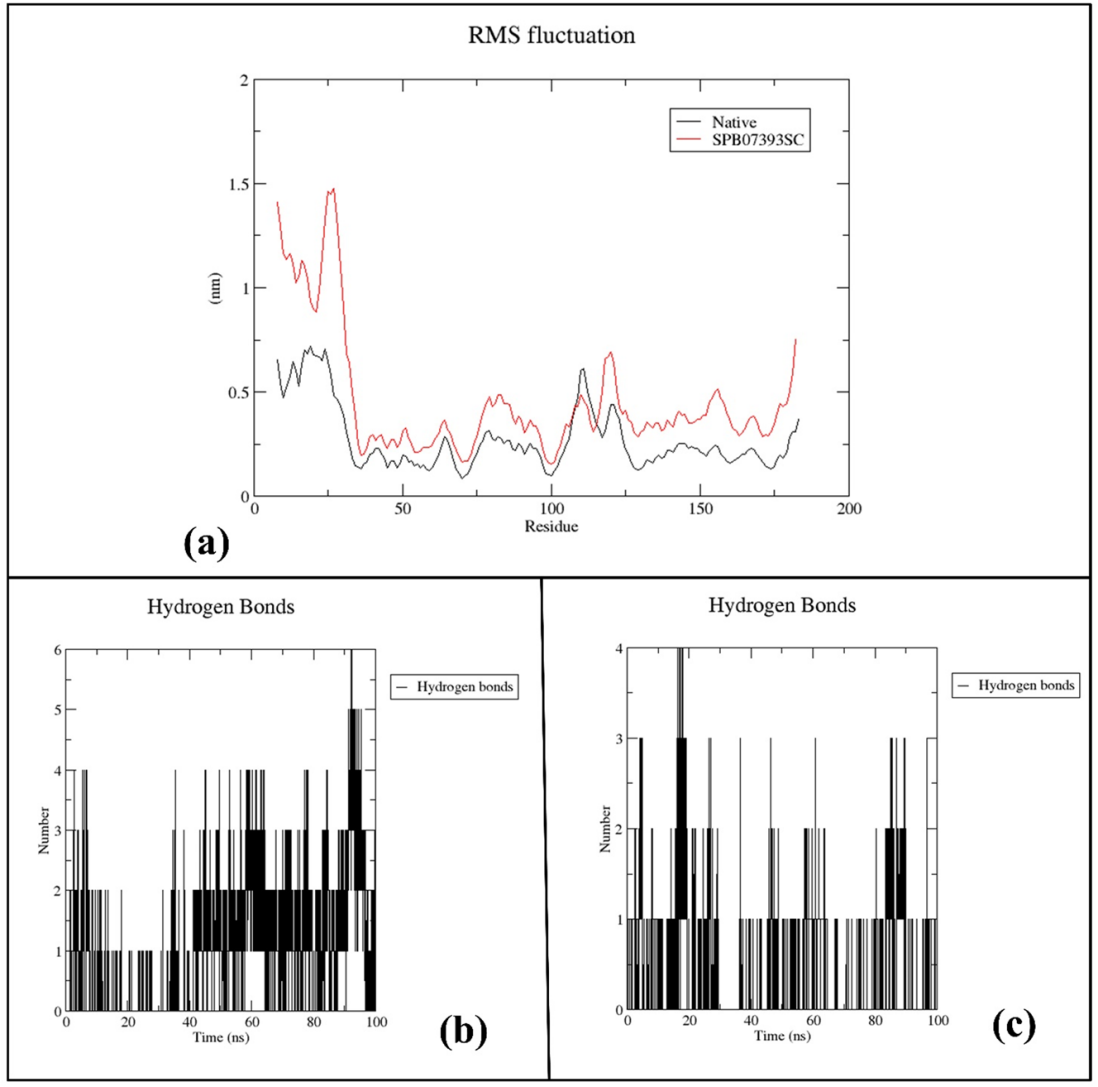
Structural dynamics calculated during the 100 ns of MD trajectories; (RMSF) of protein backbone (**a**), number of H-bonds formed with Native ligand (**b**), and **SPB07393SC** (**c**).

The RMSF analysis of backbone residues assessed their flexibility or rigidity during the 100 ns MD simulation. Both complexes showed similar RMSF patterns (Fig. 11a), with residues involved in ligand interactions exhibiting minimal fluctuation (< 0.2 nm). To assess interaction stability, we quantified the number of hydrogen bonds formed during the simulation (Fig. 11b and c). The co-crystallized ligand consistently formed 2–4 hydrogen bonds, occasionally increasing by 2 more. **SPB07393SC** formed an average of 2–3 hydrogen bonds, indicating notable stability consistent with both RMSD and Rg analyses.

Stability and flexibility properties of the protein-ligand complex were obtained from MD simulations. Although **SPB07393SC** showed high initial fluctuation, it stabilized, indicating that the ligand binds well to Glo-I. The simulation data confirm that **SPB07393SC** maintains efficient interactions with the enzyme’s active site. The ligand’s unique structural features, such as the triazole and coumarin rings, may stabilize the enzyme-ligand complex through coordination to zinc.

### In vitro enzyme bioassay

**Fig. 12:**
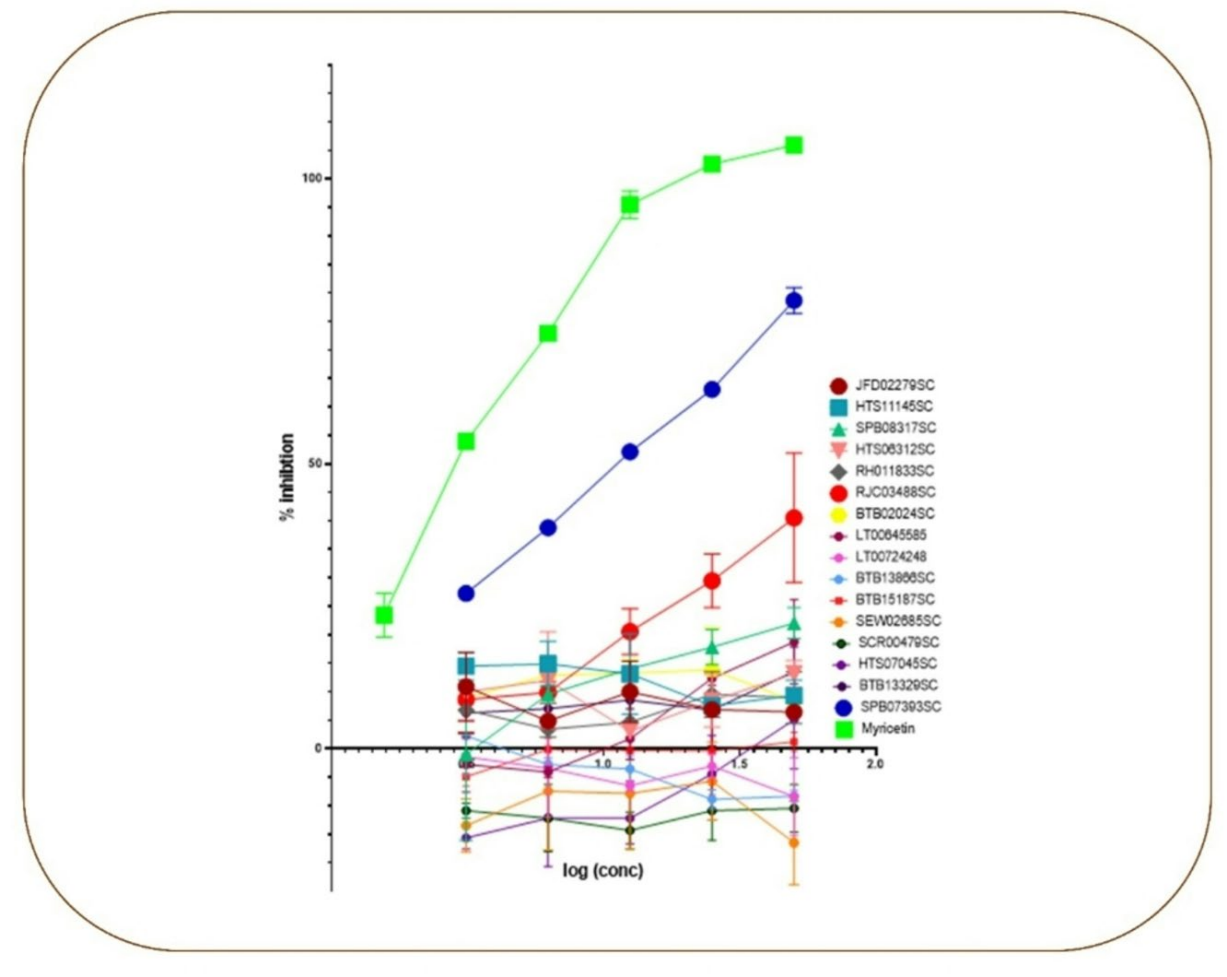
Graph showing the inhibitory activity of selected compounds (1–16) against Glo-I and their corresponding IC50 values.

The inhibitory activity of the chosen hits against recombinant human Glo-I (rhGlo-I) was tested in vitro. (Al-Balas et al., 2016) (A Al-Balasa et al., 2017). Three consecutive tests were carried out in triplicate for each selected hit using a plate reader, and the average of these data was calculated. *GraphPad Prism* 8 (2019) was used to compute the IC50 values of all selected hits. The results are given as percentages of inhibition, where the Glo-I activity of compounds 1–16 is shown. Compound 13 was found to be the most potent of the selected compounds, with an average inhibitory activity of 78.66% at 50 µM, as shown in Table 1. Compound 13 has an IC50 of (11.1µM); however, the IC50 of the remaining compounds cannot be determined as they showed inhibitory activity lower than 50% at 50µM, as shown in Fig. 12.

### Correlation between the computational and in vitro results

Although docking results displayed compound 1 (HTS06312SC) as the most active ligand (Table 1). However, in vitro inhibition of compound 13 (**SPB07393SC**) was found to be the most effective against Glo-I enzyme Fig. 8. The activity of compound 13 (**SPB07393SC**) may be a result of the compound having a hydroxyl group at the triazole ring, which could coordinate a zinc ion and generate a stable coordination complex within the active area of Glo-I. In the meantime, the para-chlorobenzene ring fills the enzyme’s hydrophobic pocket and interacts with the surrounding hydrophobic residues. These interactions stabilize the compound’s binding to the enzyme. Furthermore, the lactone ring in (**SPB07393SC**) forms a dipole interaction with polar residues on the enzyme’s surface. This interaction can enhance the compound’s binding affinity for the enzyme, thereby increasing its activity. In conclusion, the compound’s Y-shaped structure is thought to be a key factor in its activity, as it enables efficient interaction with the enzyme by optimizing binding. However, further investigation is needed to optimize the compound and to increase its activity.

The inactive Compound 1 (HTS06312SC), which is shown in Fig. 6, could be due to the following reasons: one of the main drawbacks behind the low activity for this compound could be due to its high polarity, particularly the presence of two carboxylic acid groups in the structure (high desolvation energy). The second drawback of molecular docking is its inability to imitate the real protein movement. CDocker and other docking programs utilize a rigid docking approach, which assumes that the protein is rigid. However, the protein is dynamic and may undergo conformational changes upon ligand binding, which can lead to inaccurate predictions. Furthermore, the original co-crystallized ligand inside the active site may affect the conformation of the protein and, as a consequence, the binding mode of other ligands. Also, CDocker performance can be influenced by a variety of factors, including parameter selection and the suitability of the method for the specific target protein. In the case of Compound15 (BTB02024SC) shown in (Fig. 7), we intentionally selected a compound with a positively ionized functional group (morpholine) to see if it would be active against GLO-1. As expected, we found that the compound was inactive, most likely because the positively charged group repels the amino acids at the entrance to the active site, thereby preventing the compound from entering and binding effectively. This result confirms our theory that the positively charged group would be repelled by the active site, thereby negating activity.

The disappointing results stem from several factors that require analysis to prevent future errors.

First, docking studies are often performed using a rigid receptor. In reality, enzymes are flexible molecules that can undergo conformational changes upon ligand binding. Therefore, docking studies may not fully capture the dynamic nature of ligand-receptor interactions. This may result in inaccurate predictions of binding affinity and inhibitory activity.

Second, the software has generated many tautomers and isomers to mimic real compounds, even when they are present at low levels. These generated structures may not properly reflect reality, and many tautomers and isomers may be rare or absent. This can lead to errors in selecting new candidates, particularly affecting the types of compounds chosen in subsequent steps.

Third, as a consequence, most of the compounds selected in this run have low log P values (polar). Such polarity requires a high desolvation energy, which the binding enthalpy cannot compensate for.

## Conclusions

This work presented **SPB07393SC** as a potent Glo-I inhibitor and provided valuable insights into the discovery of new chemotypes targeting this metalloenzyme. The hydroxytriazole moiety introduces a zinc-coordinating feature not described in any previous Glo-I inhibitor design. The compound shows in vitro inhibitory activity, with favorable predicted pharmacokinetic-pharmacodynamic properties and stable binding behavior. This profile underscores its potential as a strong lead for further refinement. This study not only identifies a new scaffold but also highlights broader implications for the development of Glo-I inhibitors. The discrepancies between docking predictions and experimental results highlight the limitations of static, rigid-receptor methods. They emphasize the importance of molecular dynamics and experimental verification when assessing ligand behavior at the enzyme active site. This knowledge supports more sophisticated and inclusive modeling strategies for future drug discovery.

The findings described here increase understanding of zinc-binding interactions in the Glo-I catalytic region. They also provide new ways to develop more advanced inhibitors. **SPB07393SC** may be a promising hit molecule for further optimization to improve potency, selectivity, and therapeutic potential in anticancer drug development.

## Data Availability

Data that support the findings of this study are available upon reasonable request from the corresponding author [M.B.A].
